# The relationship between personality profile and self-care among patients with type 2 diabetes

**DOI:** 10.3389/fpsyg.2022.1030911

**Published:** 2022-11-15

**Authors:** Zahra Dadras, Behnam Molaei, Masoumeh Aghamohammadi

**Affiliations:** ^1^Department of Medical-Surgical Nursing, School of Nursing and Midwifery, Ardabil University of Medical Sciences, Ardabil, Iran; ^2^Department of Family Health, Social Determinants of Health Research Center, Ardabil University of Medical Sciences, Ardabil, Iran; ^3^Department of Psychiatry, Faculty of Medicine, Ardabil University of Medical Sciences, Ardabil, Iran; ^4^Department of Emergency Nursing, School of Nursing and Midwifery, Ardabil University of Medical Sciences, Ardabil, Iran

**Keywords:** self-care behaviors, personality profile, type 2 diabetes, personality disorder, correlational study

## Abstract

**Background:**

As a chronic disease, diabetes needs special self-care behaviors until the end of life. Personality traits are considered to be effective psychological factors in controlling diabetes and self-care in patients with diabetes. The present study was conducted to determine the relationship between personality profile and self-care among people with type 2 diabetes.

**Methods:**

In this descriptive-correlational study conducted in 2021, 160 patients with type 2 diabetes referred to the diabetes clinic of Imam Khomeini Educational and Medical Center in Ardabil were selected by convenience sampling method. The data collection tools included the Diabetes Self-Care Activities questionnaire (SDSCA) and the short form of the Millon Multi-Axis Clinical Test (MCMI-3), which were completed through interviews with patients. Data were analyzed by SPSS software using descriptive statistics (mean, SD, and frequency) and inferential statistics (Pearson correlation coefficient and linear regression).

**Results:**

Based on the results, apart from the obsessive personality disorder, which had a positive relationship with self-care behaviors, a significant negative correlation was observed between schizoid, avoidant, depressed, dependent, antisocial, self-harming, borderline, and paranoid personality disorders with self-care behaviors (*p* < 0.01).

**Conclusion:**

The results showed that there is a significant negative relationship between personality profile and self-care status of patients with type 2 diabetes. In other words, a person’s personality profile can predict self-care behaviors. Accordingly, personality traits can be considered as one of the influencing factors on self-care in the educational programs of diabetic patients. Holding educational classes to empower patients using psychological interventions and teaching effective solutions can be an effective step toward increasing the level of mental-physical health and self-care of patients with type 2 diabetes.

## Introduction

Diabetes Mellitus is one of the chronic diseases that has plagued mankind for many years and is seen in all ages (Molavi, 2010). Today, diabetes is spreading worldwide as an unprecedented epidemic, and it is estimated that more than 350 million people will suffer from diabetes by 2030 (Zheng et al., 2018a). In Iran, according to the World Health Organization (WHO), the prevalence of type 2 diabetes is 10.3%, and it is reported as one of the 10 main causes of death in the country (Zangeneh et al., 2018). The two main types of diabetes are type 1 and type 2. Type 2 diabetes accounts for 85–90% of all diabetes, and its prevalence has grown significantly in recent years due to lifestyle changes and increasing obesity (American Diabetes Association, 2018).

Due to its chronic nature, diabetes affects all aspects of a person’s life, and the treatment goals mostly have to be individualized (Nanayakkara et al., 2018); thus, the most important method to manage type 2 diabetes is self-care (Henderson, 2010). Diabetes self-care is defined as a set of behaviors (setting a meal plan, exercising, taking medications, self-monitoring of blood or urine sugar levels, and foot care) that patients with diabetes perform on a daily basis to manage their diabetes (Rahimian-Boogar et al., 2010). These behaviors prevent the early and late complications of the disease and increase the life expectancy of the patients (Adams et al., 2003).

Most patients with diabetes do not consistently perform self-care measures such as diet, regular medication monitoring, and exercise (Whitebird et al., 2018; Zheng et al., 2018b). According to the studies, personality traits are one of the most effective factors in predicting self-care behaviors and controlling type 2 diabetes in people with this disease (Watson and Pennebaker, 1989; Mahmoud Alilou et al., 2014; Yasui-Furukori et al., 2020; Willroth et al., 2021).

Since the psychological aspect of diabetes is considered an important part of the treatment and control of this disease in the modern world, it seems that people with type 2 diabetes suffer from certain psycho-behavioral and personality characteristics due to factors such as dietary restrictions, movement restriction, aggressive blood sugar control, daily insulin injections, chronic physical symptoms, and frequent hospitalizations, all of which affect the process of disease control and care (Diderichsen and Andersen, 2019). According to studies, personality disorders such as depression are common in patients with diabetes, and their rate is twice that of non-diabetic individuals (Emre et al., 2018; Khaledi et al., 2019). Hysteria and hypochondria are also associated with uncontrolled type 2 diabetes (Weinstein et al., 2019). Hackinger et al. (2018) also showed that diabetes is more common in patients with schizophrenia and that schizophrenia itself can increase the risk of diabetes. Moreover, it seems that there is a significant relationship between the type of diabetes treatment and psychological problems, so that patients who use insulin to control their disease experience more complications (Hope et al., 2018). Meanwhile, the simultaneity of symptomatic diabetes and psychological disorders causes aggravation of disease symptoms, the occurrence of various side effects, reduced response to treatment, increased costs, increased mortality (Schmitt et al., 2018; Palinkas et al., 2019), poor quality of life, non-cooperation with treatment, and inappropriate use of medical services (Willroth et al., 2021). Thus, it seems necessary to identify the personality characteristics of type 2 diabetes patients.

Despite the rise in the prevalence of type 2 diabetes and the importance of medical and psychological aspects for its management, the required efforts to control this disease are often focused solely on medical care and not on the effective psychological factors (Malekmahmoodi et al., 2020). On the other hand, significant inconsistencies can be seen in the research conducted in this field, as most studies have focused on the role of one or more personality factors in adapting to diabetes. However, there are only a few studies investigating the personality profile of diabetic patients. Therefore, this study was conducted to determine the relationship between self-care and the personality profile of people with type 2 diabetes.

## Materials and methods

This was a descriptive-correlational study and was conducted in 2021. The statistical population included all patients with type 2 diabetes who were referred to the diabetes clinic of Imam Khomeini Educational and Medical Center in Ardabil. In this research, the statistical population was 6,000 people, and Cochran’s formula was used to determine the sample size. Based on Cochran’s formula for calculating the sample size and considering *p* = 0.11, the number of samples selected for the present study was 146 people, and with a probability of 10% attrition, the final sample number was estimated to be 160 people. Samples met the inclusion criteria (at least 1-year history of diabetes, clinical diagnosis of type 2 diabetes based on the laboratory tests included in the patient’s health record, absence of acute and chronic mental illnesses, absence of pregnancy, and willingness to participate in the study) and were selected by convenience sampling method.

The demographic and disease information form (gender, age, education, marital status, income level, employment status, duration of diabetes, history of diabetes in relatives, body mass index, and glycosylated hemoglobin), Tolbert & Glasgow Summary of Diabetes Self Care Activities (SDSCA) questionnaire, and Millon Clinical Multiaxial Inventory (MCMI-III) were used to collect data.

The SDSCA questionnaire is used to examine the self-care activities of patients with diabetes, such as diet compliance, blood sugar monitoring, physical activity, medication adherence, and foot care. In this questionnaire, patients were asked to state the number of days in the past week that they followed self-care behaviors. The mentioned brief questionnaire contains 15 questions in 5 areas; each area is graded on a Likert scale from not at all (0) to every day of the week (Rahimian-Boogar et al., 2010). The overall average score of self-care is calculated by dividing the total number of points obtained by 14. Higher scores indicate higher degrees of adherence to self-care behaviors. The validity and reliability of this tool have been confirmed in previous studies, and its Cronbach’s alpha coefficient has been estimated to be 0.68 (Morowati and Rouhani, 2009). In the present study, the reliability of this questionnaire was calculated by Cronbach’s alpha (α = 0.85).

Millon Clinical Multiaxial Inventory-III was designed by Millon (1985) and included 175 short self-describing sentences with yes and no answers, which is used for adults 18 years and older, with 14 clinical personality patterns and 10 clinical symptoms. The range of scores in Millon’s personality and clinical symptoms scales is from 0 to 115, and the raw score becomes the baseline score (Millon, 1985). One of the important features of the MCMI and its revised versions is the baseline rate (BR) score to confirm the presence or absence of a particular trait (the BR score, like the well-known T score, is actually a means of converting a raw score into a meaningful score for interpretation and increases the accuracy of diagnosis). Millon defined a BR score of 85 or higher as the definite presence of a personality disorder. A BR score of 75 to 85 indicates patterns and personality styles, and scores below 75 indicate the presence of some features of a disorder. Also, the score of 60 is determined as the median score of psychiatric populations, and the score of 35 is determined as the median score of normal or non-psychiatric groups. Dadfar and Lester (2017) confirmed the validity and reliability of this tool in 2012 (Dadfar and Lester, 2017). In the present study, the alpha coefficient of the scales was obtained in the range of 0.82 (schizoid personality disorder) to 0.89 (narcissistic personality disorder).

The research was carried out after receiving the ethics code from the Ethics Committee of Ardabil University of Medical Sciences and a letter of introduction from the research vice-chancellor of the faculty. The researcher provided the necessary explanations to the patients regarding the objectives of the research. Subsequently, the questionnaires were given to the patients with type 2 diabetes, and they were assured about the confidentiality of the information. Before conducting each interview regarding the objectives of the study, the confidentiality of the recorded information, the approximate duration of the interview and permission to take notes of the interview by the researchers, consent was obtained from the participants. The participants had the right to withdraw from the interview at all stages. After obtaining the informed verbal consent of the people to participate in the interview, a number was assigned to each of the participants in order to prevent the disclosure of their names, and then the interview was conducted. Finally, the data was coded and entered into SPSS software, and descriptive (mean and SD) and inferential analysis (independent *t*-test, one-way analysis of variance, linear regression model, and Pearson’s correlation coefficient test) were performed with a significance level of *p* < 0.01.

## Results

Out of the 160 patients, 61.88% were male, and 75.63% were married. Most of the samples had a high school diploma (38.12%) and were in the age range of 49–69 years (69.4%). About 36% of the patients had a body mass index higher than 25, and 98 people (61.25%) had HbA1c less than 8.5.

Regarding the state of self-care, the results showed that the average score of self-care was 32.73 ± 12.62. Also, the mean scores of blood sugar test, physical activity, general foot care, following a healthy diet, and consumption timeliness of medicines were 3.44 ± 3.84, 4.78 ± 4.25, 4.96 ± 4.93, 13.74 ± 4.31 were 6.14 ± 1.39 (Table 1).

**TABLE 1:** Mean and standard deviation of self-care scores and its dimensions.

Variable	Mean	SD
Following a healthy diet	13.74	4.31
Physical activity	4.78	4.25
Medication compliance	6.14	1.39
General foot care	4.96	4.93
Blood sugar test	3.44	3.84
Average score of self-care	32.73	12.62

The results related to the personality profile of the patients showed that the dramatic personality patterns had the highest score (65.05 ± 28.29), and the sadistic personality had the lowest score (13.20 ± 14.96) (Table 2).

**TABLE 2:** Mean and standard deviation of personality profile scores in patients with type 2 diabetes.

Personality profile	Mean	SD
Schizoid	40.80	15.77
Avoidant	30.85	17.83
Depressed	36.85	29.99
Dependent	22.80	19.56
Dramatic	65.05	28.29
Narcissistic	41.15	13.75
Antisocial	18.25	14.48
Sadistic	13.20	14.96
Obsessive	62.95	13.55
Negative	40.12	20.74
Self-injurious	26.55	21.51
Schizotypal	27.80	21.89
Borderline	25.80	20.89
Paranoid	38.40	18.88

The results of Pearson’s correlation test showed a negative and significant relationship between personality profile and self-care status of patients with type 2 diabetes (*r* = −0.38, *p* ≥ 0.01) (Table 3).

**TABLE 3:** Relationship between self-care status and personality profile of patients with type 2 diabetes.

Variables	Personality profile	
	*r*	*P*-values
Self-care	−0.38	0.01

The results showed that there is a significant negative relationship between schizoid, avoidant, depressed, dependent, antisocial, negative, self-injurious, borderline, and paranoid personality disorders with self-care dimensions. Also, there was a significant positive relationship between obsessive personality disorder and all dimensions of self-care behavior. However, no significant correlation was found between dramatic, narcissistic, sadistic, and schizotypal personality disorders with self-care dimensions (Table 4).

**TABLE 4:** Relationship between self-care dimensions and personality profile.

Variables	Following a healthy diet	Physical activity	Medication compliance	General foot care	Blood sugar test
**Schizoid**					
*r*	–0.355	–0.442	–0.311	–0.394	–0.386
*p*-values	0.008	0.001	0.001	0.003	0.01
**Avoidant**					
*r*	–0.387	–0.312	–0.352	–0.324	–0.335
*p*-values	0.003	0.001	0.001	0.001	0.01
**Depressed**					
*r*	–0.235	–0.301	–0.297	–0.285	–0.331
*p*-values	0.001	0.001	0.001	0.003	0.01
**Dependent**					
*r*	–0.244	–0.241	–0.269	–0.314	–0.218
*p*-values	0.008	0.001	0.001	0.003	0.01
**Dramatic**					
*r*	–0.145	–0.028	–0.031	–0.012	–0.045
*p*-values	0.008	0.001	0.001	0.003	0.01
**Narcissistic**					
*r*	–0.029	–0.033	–0.048	–0.028	–0.027
*p*-values	0.07	0.001	0.001	0.161	0.001
**Antisocial**					
*r*	–0.415	–0.282	–0.294	–0.398	–0.291
*p*-values	0.001	0.579	0.001	0.001	0.730
**Sadistic**					
*r*	–0.227	–0.042	–0.025	–0.027	–0.039
*p*-values	0.001	0.001	0.730	0.001	0.001
**Obsessive**					
*r*	0.415	0.494	0.425	0.488	0.490
*p*-values	0.008	0.001	0.001	0.003	0.01
**Negative**					
*r*	–0.394	–0.227	–0.384	–0.217	–0.309
*p*-values	0.003	0.001	0.001	0.001	0.01
**Self-injurious**					
*r*	–0.234	–0.306	–0.298	–0.275	–0.387
*p*-values	0.001	0.001	0.001	0.003	0.01
**Schizotypal**					
*r*	–0.018	–0.024	–0.026	–0.031	–0.021
*p*-values	0.008	0.001	0.001	0.003	0.01
**Borderline**					
*r*	–0.321	–0.311	–0.365	–0.295	–0.324
*p*-values	0.07	0.001	0.001	0.161	0.001
**Paranoid**					
*r*	–0.411	–0.325	–0.295	–0.384	–0.325
*p*-values	0.001	0.579	0.001	0.001	0.730

## Discussion

The aim of the present study was to determine the relationship between self-care and the personality profile of patients with type 2 diabetes who were referred to the diabetes clinic of Imam Khomeini Medical Education Center in Ardabil city in 2021. The findings of the current research regarding the self-care status of the patients participating in the study showed that the lowest self-care scores were in the field of blood sugar testing, physical activity, and general foot care, and the highest scores were in the areas of following a healthy diet and medication compliance. In addition, the self-care status of patients was generally not favorable, which is consistent with the results of other similar researches (Sloven, 2010; Daily, 2011; Anbari et al., 2012; Parham et al., 2014; Lin et al., 2016; Mahdilouy and Ziaeirad, 2019). Jordan (2012) also assessed the self-care behaviors of Filipino-American adults with type 2 diabetes as unfavorable. In addition, the lowest self-care activity was seen in the two dimensions, i.e., “daily blood sugar control” and “regular performance of physical activities.” The results of Lin et al. (2016) also showed that the self-care behavior of diabetic patients in the field of foot care was weak, and most of the research samples were in an unfavorable state regarding self-care behaviors. On the other hand, the studies conducted by Bageri et al. (2015), Carmen (2013), and Moeini et al. (2016) showed that the majority of patients with diabetes were at a good level in terms of various dimensions of self-care. Hosseini et al. (2016) also reported the favorable self-care ability of patients with diabetes. It seems that the variability in the self-care status of patients in the current studies is due to the difference in the self-care education programs for diabetic patients, the difference in the amount of knowledge and attitude toward self-care, as well as the difference in the measurement of self-care (ShahabJahanlo et al., 2008; Moeini et al., 2016; Ranjbaran et al., 2020). For example, in the study performed by Hosseini et al. (2016), only women constituted the research samples, while this study examined both men and women. In addition, Khosravan et al. (2015) only included diabetic women who had peripheral neuropathy and psychiatric disorders; however, we excluded diabetic patients with psychiatric disorders from our study. Also, in the study conducted by Parham et al. (2014), the data collection tool was a researcher-made questionnaire; however, in our study, the standard scale of self-care behavior of Toobert et al. (2000) was used.

The findings of the current research showed that there is a significant negative relationship between personality profile and self-care status of patients with type 2 diabetes. The findings are consistent with the studies accomplished by Pollock and Davis (2005), Sterman et al. (1981), Eun and Zeinberg (1989), Quan et al. (2020), Banan (2007), and Wiseman and Bulker (2021). The results obtained by Pollock and Davis showed that there is a relationship between demonstrative, obsessive, and borderline personality traits and self-care behaviors such as medication compliance and insulin use in diabetic patients. Also, the results showed that borderline personality traits significantly predict patients’ self-care behavior such as foot care (Pollock and Davis, 2005). Quan et al. also showed that patients with schizoid, paranoid, dramatic, antisocial, and depressed personality traits do not perform self-care measures such as diet, regular medication monitoring, and exercise well. The results showed that the coexistence of symptomatic diabetes and personality disorders causes the exacerbation of disease symptoms, reduction of response to treatment, increase in costs, and poor self-care of patients (Quan et al., 2020). Despite the above studies, in some studies, there was no significant relationship between self-care behaviors and personality disorders of patients with diabetes (Jasper, 1989; Phillips, 2012; Norton, 2014; Morgan, 2022). The results of Morgan (2022) showed that there is a correlation between the personality disorder of patients and self-care behaviors such as foot care and blood sugar monitoring, and there is no significant relationship with medication compliance. It seems that the reason for the difference in the results related to the relationship between self-care status and the personality profile of patients with diabetes in different countries and cultures is due to the difference in sampling methods, the type of tools used, and different interview techniques.

In the regression analysis of self-care behaviors, the β value of the variables including avoidant (−0.245), depressed (−0.234), dependent (−0.476), antisocial (−0.704), obsessive (0.500), negative oriented (−0.571), self-harm (−0.313), schizotypal (−0.503), and borderline (−0.146) were significant, and these variables predicted self-care behaviors in patients with type 2 diabetes (Table 5).

**TABLE 5:** Results of regression analysis of predicting self-care behaviors by personality profile.

*P*-value	*B*	SE	β	*t*	*P*-value
Constant value (a)	93.771	16.511	–	5.679	0.000
Schizoid	–0.999	0.912	–0.241	–1.095	–0.281
Avoidant	–1.211	1.030	–0.245	–1.176	–0.048
Depressed	–2.114	1.842	–0.234	–1.148	–0.015
Dependent	–3.380	1.255	–0.476	–2.692	–0.011
Dramatic	–0.268	1.735	–0.036	–0.154	0.878
Narcissistic	0.283	0.947	–0.067	0.299	0.767
Antisocial	–3.394	0.894	–0.704	–3.795	–0.001
Sadistic	0.114	0.961	0.026	0.118	0.907
Obsessive	2.089	0.897	0.500	0.329	0.026
Negative	–3.156	1.018	–0.571	–3.110	0.004
Self-injurious	–1.426	1.110	–0.313	–1.555	–0.029
Schizotypal	–3.699	1.521	–0.503	–2.433	0.020
Borderline	–2.111	1.538	–0.146	–1.373	0.017
Paranoid	–0.220	1.046	–0.046	–0.210	0.835

The results showed that there is a significant negative relationship between schizoid personality disorder and self-care dimensions. The findings are consistent with the studies of Pollock and Davis (2005), Sterman et al. (1981), and Quan et al. (2020). Sterman et al. (1981) showed that there is a significant negative relationship between schizoid personality disorder and self-care behaviors such as medication compliance and insulin injection in type 2 diabetes patients. Quan et al. (2020) also showed that patients with schizoid personality traits do not perform self-care measures such as diet, monitoring regular medication, and exercise well and have poor self-care. However, in studies performed by Phillips (2012) and Norton (2014), there was no significant relationship between self-care behaviors and schizoid personality disorders in patients with diabetes. Individuals with schizoid personality disorder show two patterns of separation from social relationships and a limited range of emotional expression. Schizoids are therefore isolated and prefer solitary activities and have difficulty in creating and maintaining relationships and as a result, have low self-care behaviors.

The results showed that there is a significant negative relationship between avoidant personality disorder and self-care dimensions. Sun Chang (2004) showed that avoidant personality disorder negatively affects the self-care of diabetic patients. Also, the results showed that avoidant people never blame themselves and expect others to respect all their thoughts and behaviors and accept them unconditionally (Sun Chang, 2004). Eun and Zeinberg also showed that people with avoidant personality disorder perform poorly in self-care dimensions, such as performing blood sugar tests, physical activity, and medication compliance. Moreover, the results showed that the characteristics of avoidant personality significantly predict the self-care behavior of patients such as foot care (Eun and Zeinberg, 1989). However, in the study by Morgan, there was no significant relationship between self-care behaviors such as foot care, blood sugar control, and medication compliance and avoidant personality disorders in patients with diabetes (Morgan, 2022). It seems that people with avoidant personality deliberately engage in self-care behaviors because they feel incompetent and are hypersensitive to judgment and are highly disturbed and offended when faced with it. The existence of a negative relationship between self-care and symptoms of avoidant personality disorder seems reasonable.

The results showed that there is a significant negative relationship between depressive personality disorder and self-care dimensions. Most of the studies show that patients with diabetes who have fewer self-care abilities suffer from depression (Mahdilouy and Ziaeirad, 2019). Mahmoud Alilou et al. (2014) showed an inverse relationship between depressive personality disorder and performing self-care behaviors such as diet, exercise, and self-monitoring of blood sugar. The results of the study by Khaledi et al. (2019) also indicated that depression is associated with the weakening of self-care behaviors of diabetics. In the research conducted by Lin et al. (2016), there was no significant difference in foot care and medication compliance in two groups of depressed and non-depressed diabetic patients. Depressed patients consider their situation hopeless due to lack of flexibility, and their behavioral habits including self-care behaviors, are often disturbed.

The results showed that there is a significant negative relationship between dependent personality disorder and self-care dimensions. The findings of this research are consistent with the studies of Sterman et al. (1981), Eun and Zeinberg (1989), Quan et al. (2020), and Wiseman and Bulker (2021). The results of Eun and Zeinberg (1989) showed that there is a significant negative relationship between dependent personality traits and self-care behaviors such as medication compliance and injecting insulin in diabetic patients. Quan et al. (2020) showed that there was a significant negative relationship between personality traits related to self-care measures, such as following a diet, monitoring regular medication use, and performing physical activity. While in study conducted by Jasper (1989), no significant relationship was found between self-care behaviors and dependent personality disorder in patients with diabetes. Patients with dependent personality disorder have a poor self-care due to excessive reliance on others, low self-confidence and difficulty in making decisions and the existence of a negative relationship between self-care and symptoms of dependent personality disorder seems logical.

The results showed that there is a significant negative relationship between antisocial personality disorder and self-care dimensions. A significant negative relationship has been shown between antisocial personality traits and medication compliance and insulin injection in diabetic patients (Pollock and Davis, 2005). Quan et al. (2020) also showed that patients with antisocial personality traits perform poorly in self-care behaviors such as following a healthy diet and monitoring regular medication use. In a study by Morgan (2022), there was no significant relationship between self-care behaviors and antisocial personality disorders in patients with type 2 diabetes. It can be said that patients with antisocial personality disorder have fewer self-care abilities due to impulsivity and incorrect justifications for performing desirable behaviors.

The results showed that there is a significant negative relationship between paranoid personality disorder and self-care dimensions. Banan (2007) displayed that patients who show paranoid thoughts have a negative attitude toward self-care (Banan, 2007). Additionally, another study concluded that patients with paranoid personality traits do not perform self-care measures such as diet, regular medication monitoring, and physical activity well (Quan et al., 2020). On the other hand, Jasper (1989) and Phillips (2012) reported the lack of a significant relationship between self-care behaviors, including foot care and medication compliance, with paranoid personality disorder in patients with diabetes. Paranoid personality traits have a significant effect on the mental and physical health of patients as it causes severe mental tensions, which can lead to poor self-care.

The results showed that there is a significant negative relationship between borderline personality disorder and self-care dimensions. The findings of this research are consistent with the studies conducted by Sterman et al. (1981), Eun and Zeinberg (1989), Quan et al. (2020), and Wiseman and Bulker (2021). In the research conducted by Eun and Zeinberg, people with borderline personality showed low self-care behaviors. The obtained results showed that there is a significant negative relationship between borderline personality traits and self-care behaviors such as medication compliance and injecting insulin in diabetic patients (Eun and Zeinberg, 1989). Quan et al. (2020) showed that there is a significant negative relationship between borderline personality traits and self-care measures such as the following a healthy diet, monitoring regular medication use, and doing physical activity. While Jasper (1989) expressed that no significant relationship was found between self-care behaviors and individuals with borderline personality disorder who are emotionally unstable and show irregular behavior. In explaining this finding, it can be said that borderline people think that other people cannot provide emotional support to them, and confirming their faults may lead to subsequent rejection; hence, they show more inconsistent self-care behaviors.

The results showed that there is a positive correlation between obsessive personality and self-care behaviors. The findings of Mahmoud Alilou et al. (2014) showed that there is a significant relationship between obsessive personality traits and self-care dimensions such as following a healthy diet, monitoring regular medication use, and performing physical activity. Pollock and Davis (2005) concluded that obsessive personality significantly predicts patients’ self-care behavior such as foot care and physical activity. On the other hand, the results of Morgan (2022) showed that there is no significant relationship between obsessive personality disorder and self-care behaviors such as foot care, blood sugar control, and medication compliance. In explaining the positive relationship between obsessive personality and self-care behaviors, it can be said that individuals with an obsessive personality are conscientious, over-committed to performing tasks and bound to order, and it seems logical that these people are more sensitive to self-care behaviors.

The current research has several limitations; this study was a correlational study, therefore the relationships between the variables do not indicate cause and effect relationships. Additionally, the data were collected by self-reporting and may not reflect the actual performance of individuals. Also, among other limitations of this research, we can mention the sample size and data generalization to the whole society.

## Conclusion

The results indicated a significant negative relationship between the personality profile and the self-care status of patients with type 2 diabetes. Avoidant, depressed, dependent, antisocial, obsessive, negative, self-injurious, schizotypal, and borderline personality patterns were important predictors of self-care behaviors. So, personality traits should be considered as one of the influencing factors on self-care in educational programs for diabetic patients. For this purpose, it is recommended that diabetes educators evaluate the personality characteristics of their patients before implementing the training program and adjust their care plans based on these characteristics to achieve optimal self-care behaviors in patients. According to the findings of the research, it is suggested that educational and intervention programs be organized in connection with the diagnosis and correction of some personality traits that can lead to harming the self-care of patients with type 2 diabetes for empowering the patients. It is suggested that in future studies, the research should be carried out at a wider level and field in terms of location, and the results should be compared. Choosing a sample from a larger statistical population in future surveys can help the generalization of the findings. It is suggested that future researchers use qualitative methods and techniques, such as the use of in-depth interviews and participatory observation for modeling and theorizing about the current research.

## Data availability statement

The raw data supporting the conclusions of this article will be made available by the authors, without undue reservation.

## Ethics statement

The studies involving human participants were reviewed and approved by the Ethics Committee of Ardabil University of Medical Sciences (IR.ARUM.REC.1400.239). The patients/participants provided their written informed consent to participate in this study.

## Author contributions

ZD, BM, and MA designed the study. ZD collected the data. ZD and BM analyzed the data. ZD and MA had a role in preparing the manuscript. All authors approved the final manuscript.
